# Peguero-Lo Presti criteria for the diagnosis of left ventricular hypertrophy: A systematic review and meta-analysis

**DOI:** 10.1371/journal.pone.0246305

**Published:** 2021-01-29

**Authors:** Zongying Yu, Jie Song, Li Cheng, Shasha Li, Qun Lu, Yafeng Zhang, Xiaoci Lin, Dadong Liu

**Affiliations:** 1 Department of Electrocardiography, The No. 4 Affiliated Hospital of Jiangsu University, Zhenjiang, China; 2 Department of Cardiology, The No. 4 Affiliated Hospital of Jiangsu University, Zhenjiang, China; 3 Department of Infection Management, Affiliated Hospital of Jiangsu University, Zhenjiang, China; 4 Department of Internal Medicine, The First People's Hospital of Daishan, Zhejiang, China; 5 Department of Critical Care Medicine, Affiliated Hospital of Jiangsu University, Zhenjiang, China; Albert Einstein College of Medicine, UNITED STATES

## Abstract

**Background:**

The Peguero-Lo Presti criteria are novel electrocardiographic (ECG) diagnostic criteria for the detection of left ventricular hypertrophy (LVH) and represent the sum of the amplitude of the deepest S wave in any lead with the S wave in lead V_4_ (S_D_+SV_4_). The diagnostic efficacy of the Peguero-Lo Presti criteria in LVH is still debatable. We aimed to test the sensitivity and specificity of the Peguero-Lo Presti criteria and compared them with those of the Cornell voltage index to assess their overall performance in LVH diagnosis.

**Methods:**

Electronic databases (e.g., Medline, Web of Knowledge, Embase, and the Cochrane Library) were searched from their inception until May 18, 2020. Trials written in English that investigated the Peguero-Lo Presti criteria for detecting LVH were included. Data were independently extracted and analyzed by two investigators.

**Results:**

A total of 51 records were screened, and 6 trials comprising 13,564 patients were finally included. A bivariate analysis showed that the sensitivity of the Peguero-Lo Presti criteria (0.52, 95% confidence interval (CI) 0.46–0.58) was higher than that of the Cornell voltage index (0.29, 95% CI 0.23–0.36) and Sokolow-Lyon criteria (0.24, 95% CI 0.21–0.27); the diagnostic accuracy of the Peguero-Lo Presti criteria (0.69, 95% CI 0.65–0.73) was also higher than that of the Cornell voltage index (0.67, 95% CI 0.62–0.71) and Sokolow-Lyon criteria (0.28, 95% CI 0.25–0.32); and the specificity of the Peguero-Lo Presti criteria (0.85, 95% CI 0.79–0.90) was similar to that of the Cornell voltage index (0.92, 95% CI 0.89–0.95) and Sokolow-Lyon criteria (0.94, 95%CI 0.88–0.97). Two trials (including 12,748 patients) were discharged because they included partly healthy subjects and accounted for substantial heterogeneity. Pooled analysis of the remaining 4 trials (including 816 patients) showed that the sensitivity of the Peguero-Lo Presti criteria (0.56, 95% CI 0.51–0.61) was also higher than that of the Cornell voltage index (0.36, 95% CI 0.31–0.42) and Sokolow-Lyon criteria (0.24, 95% CI 0.18–0.31); the diagnostic accuracy of the Peguero-Lo Presti criteria (0.84, 95% CI 0.80–0.87) was also higher than that of the Cornell voltage index (0.54, 95% CI 0.50–0.58) and Sokolow-Lyon criteria (0.38, 95% CI 0.34–0.42); and the specificity of the Peguero-Lo Presti criteria (0.90, 95% CI 0.87–0.92) was similar to that of the Cornell voltage index (0.93, 95% CI 0.88–0.96) and Sokolow-Lyon criteria (0.97, 95% CI 0.90–0.99). Both the likelihood ratio and posttest probability of the Peguero-Lo Presti criteria and Cornell voltage index were moderate.

**Conclusion:**

Based on this systematic review and meta-analysis, the Peguero-Lo Presti criteria-based ECG diagnostic method for LVH has high sensitivity, specificity and diagnostic accuracy and should be applied in clinical practice settings.

## Introduction

Left ventricular hypertrophy (LVH) is an important cause of arrhythmia, heart failure and sudden cardiac death [1–3]. Early diagnosis and prompt therapy might reverse the mechanism of LVH and improve the clinical outcomes of patients [4]. Electrocardiogram (ECG) is the most common diagnostic tool in the immediate screening of LVH due to its established clinical value, broad availability, and low costs. Several ECG criteria (at least 35 kinds) have been proposed in clinical practice [5–7]. However, all current criteria have poor sensitivity [8–11]. Therefore, exploring new ECG criteria with adequate sensitivity is necessary for clinical practice.

In 2017, Peguero et al. introduced a novel ECG voltage criterion (named the Peguero-Lo Presti criteria), which is the sum of the amplitude of the deepest S wave in any lead with the S wave in lead V_4_ (S_D_+SV_4_), to diagnose LVH [12]. They demonstrated that this criterion had a higher sensitivity (62% VS 35%) and similar specificity (90% VS 92%) than those in the Cornell voltage index, which is the most commonly used ECG criterion. This finding has been confirmed by many studies [13–15]. However, many scholars argue that the Peguero-Lo Presti criteria may not be a better screening tool for LVH in certain populations (obese patients and Asian populations) [16, 17]. The latest study conducted by Ricciardi et al. found that the Cornell voltage index had a more accurate diagnostic performance than the Peguero-Lo Presti criteria [18].

The most recent meta-analysis performed by Noubiap et al. proved that the Peguero-Lo Presti criteria have better diagnostic performance than the Cornell voltage index and Sokolow-Lyon criteria and might be more useful in routine clinical practice as a screening tool for LVH [19]. However, substantial heterogeneity was observed. Unfortunately, the authors did not conduct an exploratory analysis of the sources of heterogeneity. After carefully reading the study, we found that the different reference standards used for LVH diagnosis (echocardiography and cardiac MRI) and the inclusion of abstract studies may have been the main cause leading to the substantial heterogeneity.

From the above, it is still difficult to reach a consensus about the true diagnostic efficacy of the Peguero-Lo Presti criteria in LVH. Here, we conducted this systematic review and meta-analysis to assess the accuracy and clinical value of the Peguero-Lo Presti criteria of LVH to guide clinical practice and pave the way for future research.

## Methods

This meta-analysis was performed following the Preferred Reporting Items for Systematic Reviews and Meta-analyses (PRISMA) guideline recommendations (S1 Table).

### Search strategy

Two independent investigators (Zongying Yu and Qun Lu) systematically searched electronic databases (PubMed, Cochrane Library, EMBASE, and Web of Science) for studies that assessed the Peguero-Lo Presti criteria for the diagnosis of LVH from their inception until May 18, 2020. Our search terms included “Peguero-Lo Presti” and “left ventricular hypertrophy”. Electronic search terms are shown in S2 Table. The article language was restricted to English. We also checked the references in the retrieved papers to find relevant studies.

### Study selection and exclusion

Inclusion criteria: (1) All patients who underwent a 12-lead ECG and a transthoracic echocardiogram were included, regardless of their initial admission diagnosis. (2) The reference standard for the diagnosis of LVH was 2-D echocardiography (M-mode criteria) according to the American Society of Echocardiography (ASE) [20]. LVH was defined as a left ventricular mass index (LVMI) >115 g/m^2^ for male subjects and >95 g/m^2^ in female subjects. (3) Studies should provide the ECG criteria (including the Peguero-Lo Presti criteria, Cornell voltage index, and Sokolow-Lyon criteria) for the diagnosis of LVH. The Peguero-Lo Presti criteria: S_D_+SV_4_ ≥2.8 mV for male subjects and S_D_+SV_4_ ≥2.3 mV for female subjects. The Cornell voltage index: RaVL+SV_3_ >2.8 mV for male subjects,and RaVL+SV_3_ >2.0 mV for female subjects. The Sokolow-Lyon criteria: SV_1_+RV_5_/RV_6_ ≥3.5 mV. (4) Studies should provide sufficient information to calculate the sensitivity and specificity of ECG criteria for diagnosing LVH.

Exclusion criteria: (1) Review, abstract, case report, expert opinions, letter or editorials. (2) Animal or cell experiments. (3) Studies involving children or pregnant women. (4) Studies that did not provide sufficient information. (5) Duplicated publications.

All the studies were reviewed by 2 independent investigators (Jie Song and Li Cheng). Disagreements were resolved through discussion with a third investigator (Dadong Liu).

### Data extraction

Two investigators (Qun Lu and Yafeng Zhang) independently extracted data from the included studies. The data included the study characteristics (e.g., first author, publication year, sample size, study design and setting), patient characteristics (e.g., age, sex ratio, region, admitting diagnosis), and the number of true positives (TP), false negatives (FN), false positives (FP), and true negatives (TN), which were extracted using a predesigned electronic form. For any key absent information reported in the primary studies, we requested the information from the authors by e-mail. Studies were excluded if we did not obtain a response from the authors.

### Quality assessment

Two investigators (Xiaoci Lin and Dadong Liu) independently assessed the methodological quality of each eligible study by the Quality Assessment of Diagnostic Accuracy Studies (QUADAS) tool [21]. This tool included 14 questions involving patient selection, index test, reference standard, flow and timing.

### Statistical analyses

The data were analyzed using STATA v12.0 and Review Manager 5.3 statistical software by two investigators (Zongying Yu and Dadong Liu) independently.

TP, FN, FP, and TN were tabulated to calculate the sensitivity, specificity, positive likelihood ratio (PLR), negative likelihood ratio (NLR), and corresponding confidence interval (CI). The exact binomial rendition of the bivariate mixed-effects regression model was used to synthesize data. Based on this model, we calculated the mean logistic sensitivity and specificity with their standard error and 95% CIs, the between-study variability in logistic sensitivity and specificity, and the covariance between them. Based on these quantities, we constructed the summary receiver operating characteristic (SROC) curve for the ECG diagnostic criteria with summary operating points for sensitivity and specificity on the curves. *I^2^* was used to assess the heterogeneity.

## Results

### Literature search

**Fig 1:**
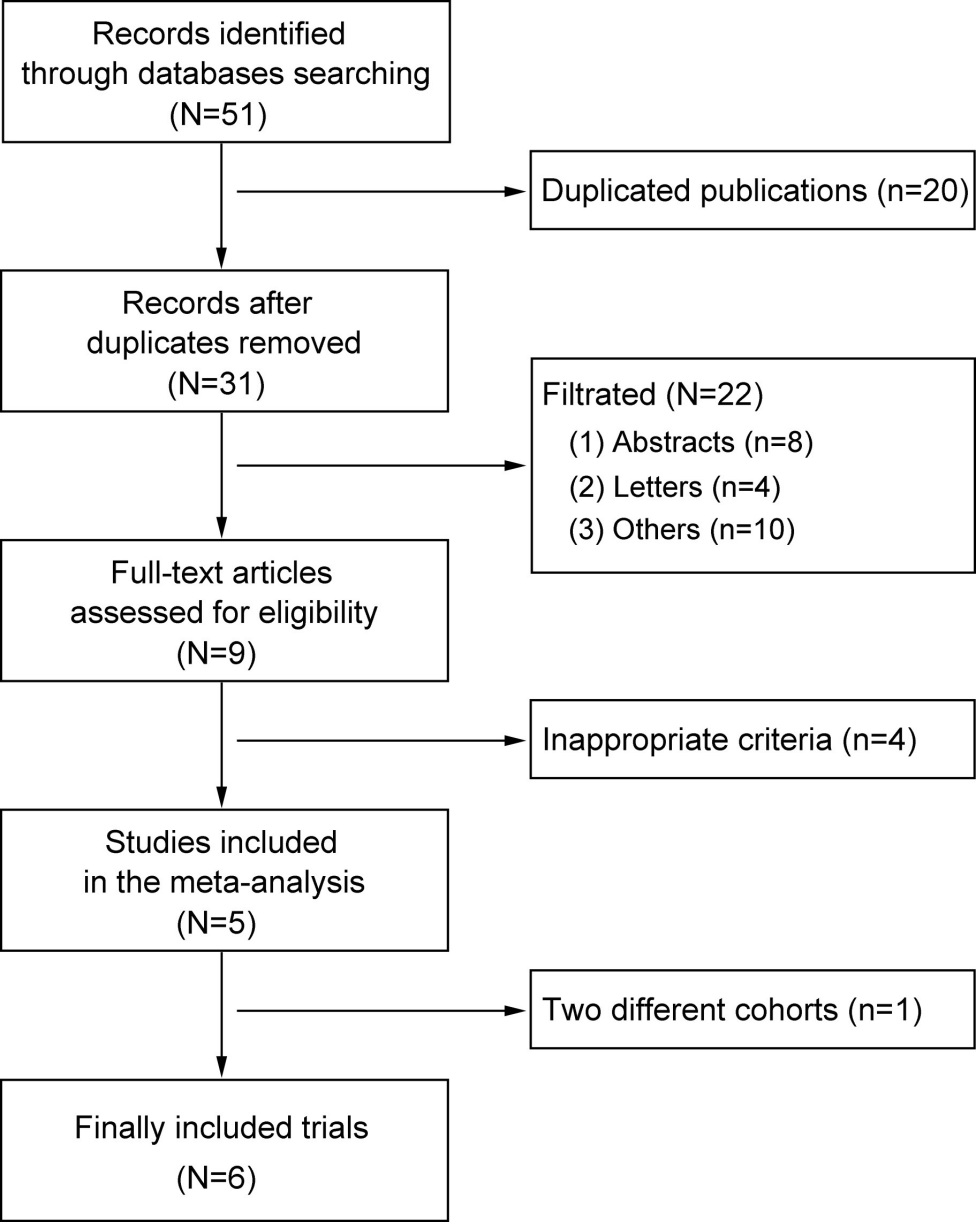
Flow diagram of the study selection process.

A total of 51 studies were identified during the initial database search. After removing duplicates, 20 studies were excluded. After the titles and abstracts were reviewed, 22 studies were excluded for different reasons, leaving 9 studies for careful review of the full text. After carefully reviewing the full text, 4 studies were excluded for inappropriate LVH diagnosis criteria [15, 17, 22, 23], leaving 5 studies to be included [12–14, 16, 18]. One study conducted by Peguero et al. had 2 different cohorts (test and validation cohorts) [12]. In total, we included 6 trials in this study. The information for primary exclusion is presented in Fig 1.

### Study characteristics

General characteristics of the included subjects are summarized in Table 1. A total of 13,564 patients (male: 6,389, female: 7,175) were included, and 7,023 (51.82%) patients had hypertension. The mean age of the included patients ranged from 53.7 to 63.79 years. Three trials reported the body mass index (BMI) [13, 16, 18]. The mean BMI of the included patients ranged from 24.80 to 31.0 kg/m^2^. Four trials reported the body surface area (BSA) [12–14]. The mean BSA of the included patients ranged from 1.67 to 2.05 m^2^. According to the echocardiography results, the prevalence of LVH among studies ranged from 10.44% to 58.62% (mean 20.02%). The included studies were conducted in Egypt [13], India [14], the USA [12], Italy [18] and China [16].

**Table 1:** Characteristics of the included subjects.

Name	Publication year	Sample size (n)	Sex ratio (M/F)	Hypertension (%)	Age (years)	BMI (kg/m^2^)	BSA (m^2^)	LVH ratio (%)	Region
Moustafa ^**13**^	2019	200	159/41	85 (42.50)	59.80±8.60	31.10±4.90	2.05±0.17	83 (41.50)	Egypt
Patted ^**14**^	2018	400	294/106	400 (100.00)	63.79±10.36	Unclear	1.67±0.10	192 (48.00)	India
Peguero^TC **12**^	2017	94	47/47	47 (50.00)	54.00±17.00	Unclear	1.91±0.27	30 (31.91)	USA
Peguero^VC **12**^	2017	122	59/63	84 (68.85)	68.00±15.00	Unclear	1.87±0.25	51 (41.80)	USA
Ricciardi ^**18**^	2020	2,134	1,014/1,120	1,033 (48.41)	69.00±13.00	25.90±4.00	Unclear	1,251 (58.62)	Italy
Sun ^**16**^	2018	10,614	4,816/5,798	5,380 (54.93%)	53.70±10.50	24.80±3.60	Unclear	1,108 (10.44)	China

TC: test cohort; VC: validation cohort; BMI: body mass index; BSA: body surface area; LVH: left ventricular hypertrophy.

Characteristics of the included trials are summarized in S3 Table. Two trials were cross-sectional studies [13, 14], and the remaining 4 trials were retrospective studies [12, 16, 18]. Four trials reported the admitting diagnosis (coronary artery disease [13], hypertension [14], and cardiovascular disease [12]) of the included patients. The histories of heart disease and medication are recorded in S4 Table. Three trials reported the history of myocardial infarction (26.50%, 10.64%, and 9.02%), the rate of coronary artery bypass graft (26.50%,10.64%, and 9.02%), and the rate of percutaneous coronary intervention (76.00%, 8.51%, and 8.20%) [12, 13]. Two trials reported the medication history [12].

### Quality of the included trials

**Fig 2:**
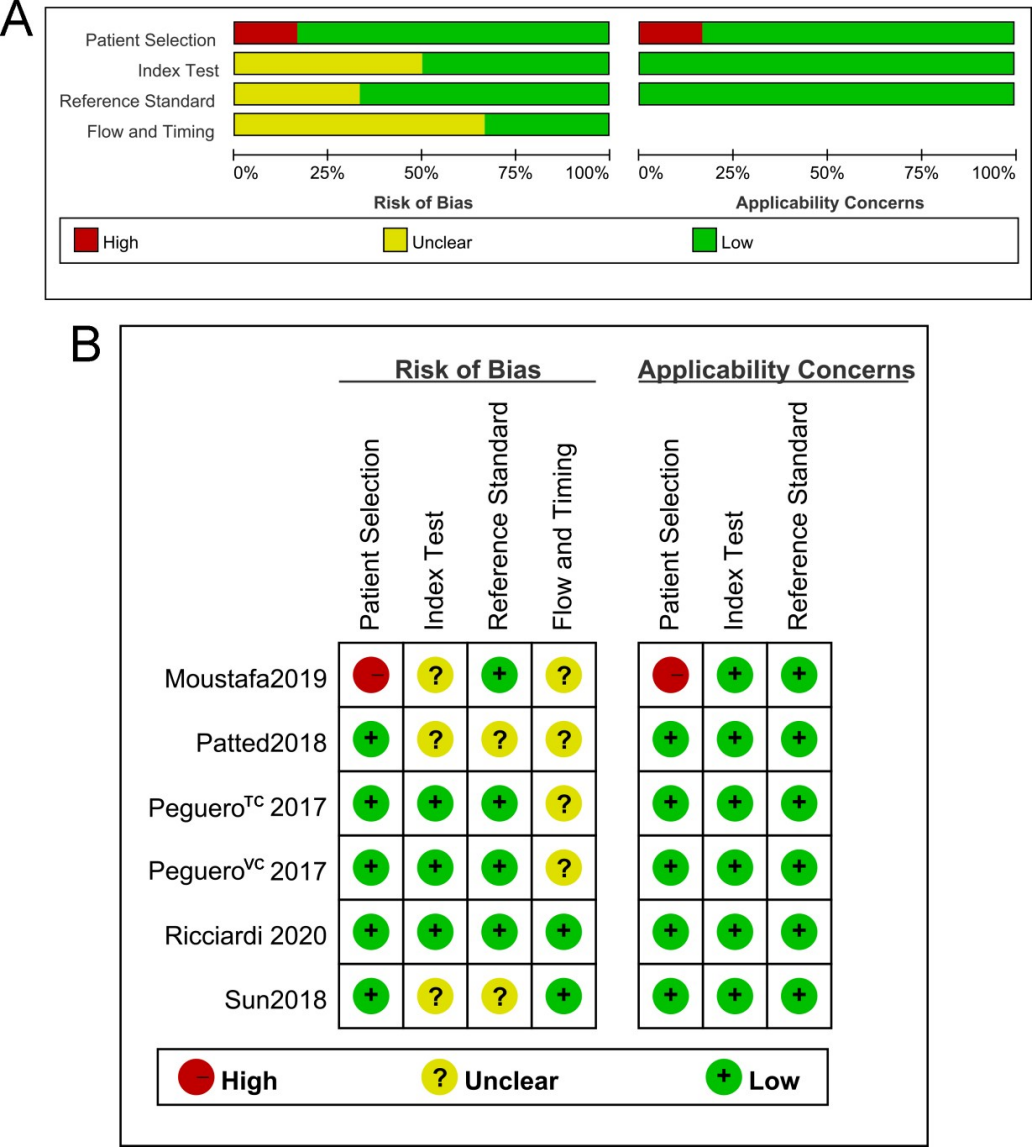
Quality of the included trials. A. Graph of the risk of bias and applicability concerns. B. Summary of the risk of bias and applicability concerns.

The methodological quality of the included studies was high (Fig 2). One study conducted by Moustafa et al. had a high patient selection bias and high applicability concerns because the included patients had been divided into 2 different groups (LVH group and no LVH group) [13]. Other biases (index test, reference standard, flow and timing) of all included studies were acceptable. All of the included studies had low applicability concerns (index test and reference standard).

### Summary of findings of the included trials

According to the echocardiography diagnosis results, the accuracies of the 3 ECG criteria for the diagnosis of LVH are summarized in Table 2.

**Table 2:** Accuracy of the ECG criteria for the diagnosis of LVH.

Name	Publication year	Sensitivity (%)			Specificity (%)		
		Peguero-Lo Presti	Cornell voltage	Sokolow-Lyon	Peguero-Lo Presti	Cornell voltage	Sokolow-Lyon
Moustafa ^**13**^	2019	55.42	32.53	26.51	87.18	97.44	98.29
Patted ^**14**^	2018	54.17	39.58	29.17	91.35	89.42	86.54
Peguero^TC **12**^	2017	70.00	40.00	23.33	89.06	90.63	96.88
Peguero^VC **12**^	2017	56.86	31.37	13.73	90.14	92.96	98.59
Ricciardi ^**18**^	2020	42.37	31.10	24.78	75.76	88.79	91.62
Sun ^**16**^	2018	47.11	19.77	23.47	75.41	95.89	88.88

TC: test cohort; VC: validation cohort; LVH: left ventricular hypertrophy.

**Fig 3:**
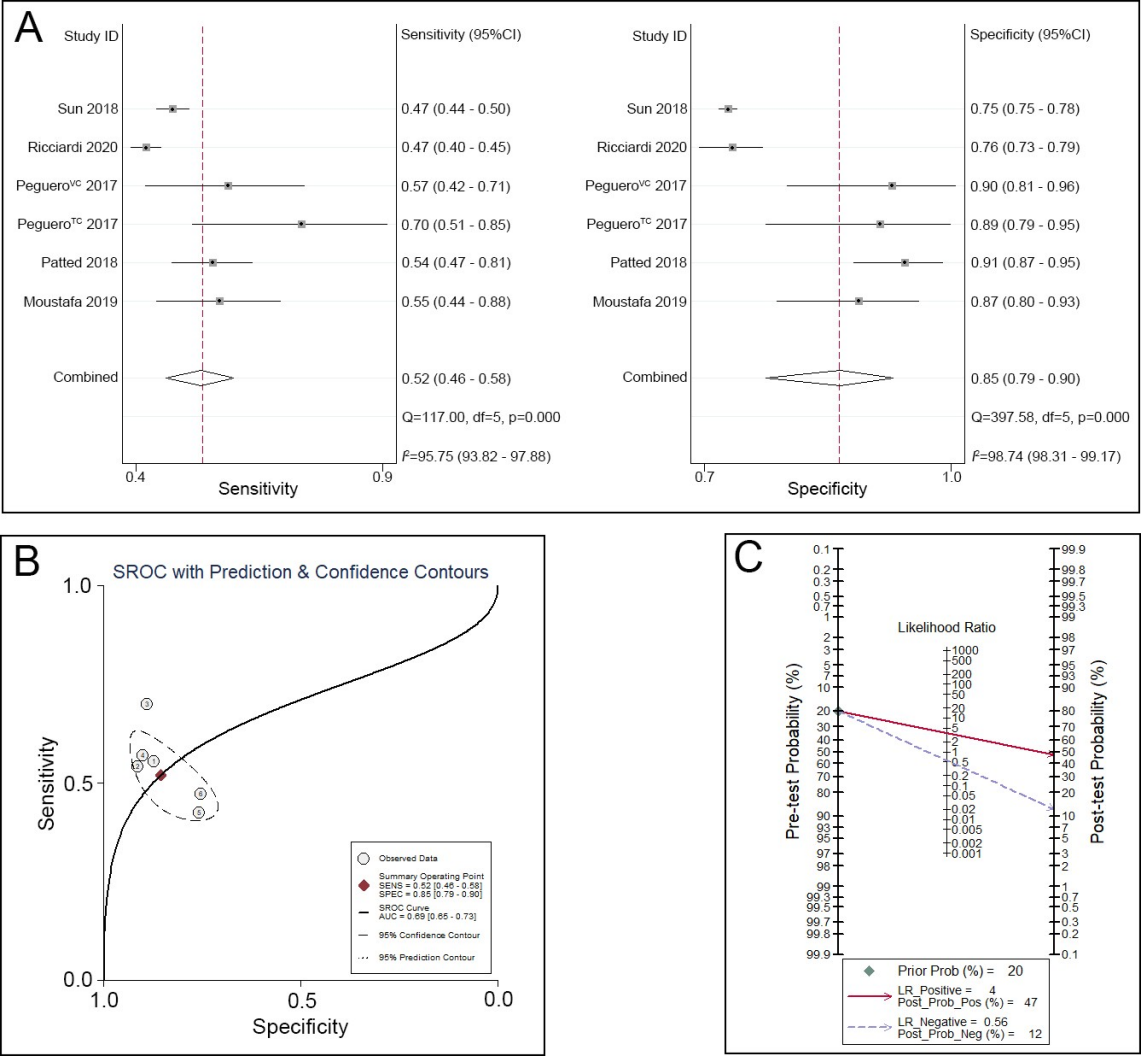
Peguero-Lo Presti criteria for the diagnosis of LVH. A. Sensitivity and specificity of the Peguero-Lo Presti criteria for the diagnosis of LVH. B. SROC curve of the Peguero-Lo Presti criteria for the diagnosis of LVH. C. Fagan nomogram of the Peguero-Lo Presti criteria for the diagnosis of LVH.

Regarding the Peguero-Lo Presti criteria, the sensitivity ranged from 0.42 to 0.70, and the specificity ranged from 0.75 to 0.91. A pooled analysis of the Peguero-Lo Presti criteria in the diagnosis of LVH showed that the pooled sensitivity was 0.52 (95% CI 0.46–0.58), the specificity was 0.85 (95% CI 0.79–0.90) (Fig 3A), and the area under the SROC curve (AUC) was 0.69 (95% CI 0.65–0.73) (Fig 3B). Given a pretest probability of 20%, the pooled PLR and NLR of the Peguero-Lo Presti criteria for the diagnosis of LVH were 4 and 0.56, respectively; the posttest probability for a positive test result was 47%, and the posttest probability for a negative test result was 12% (Fig 3C).

**Fig 4:**
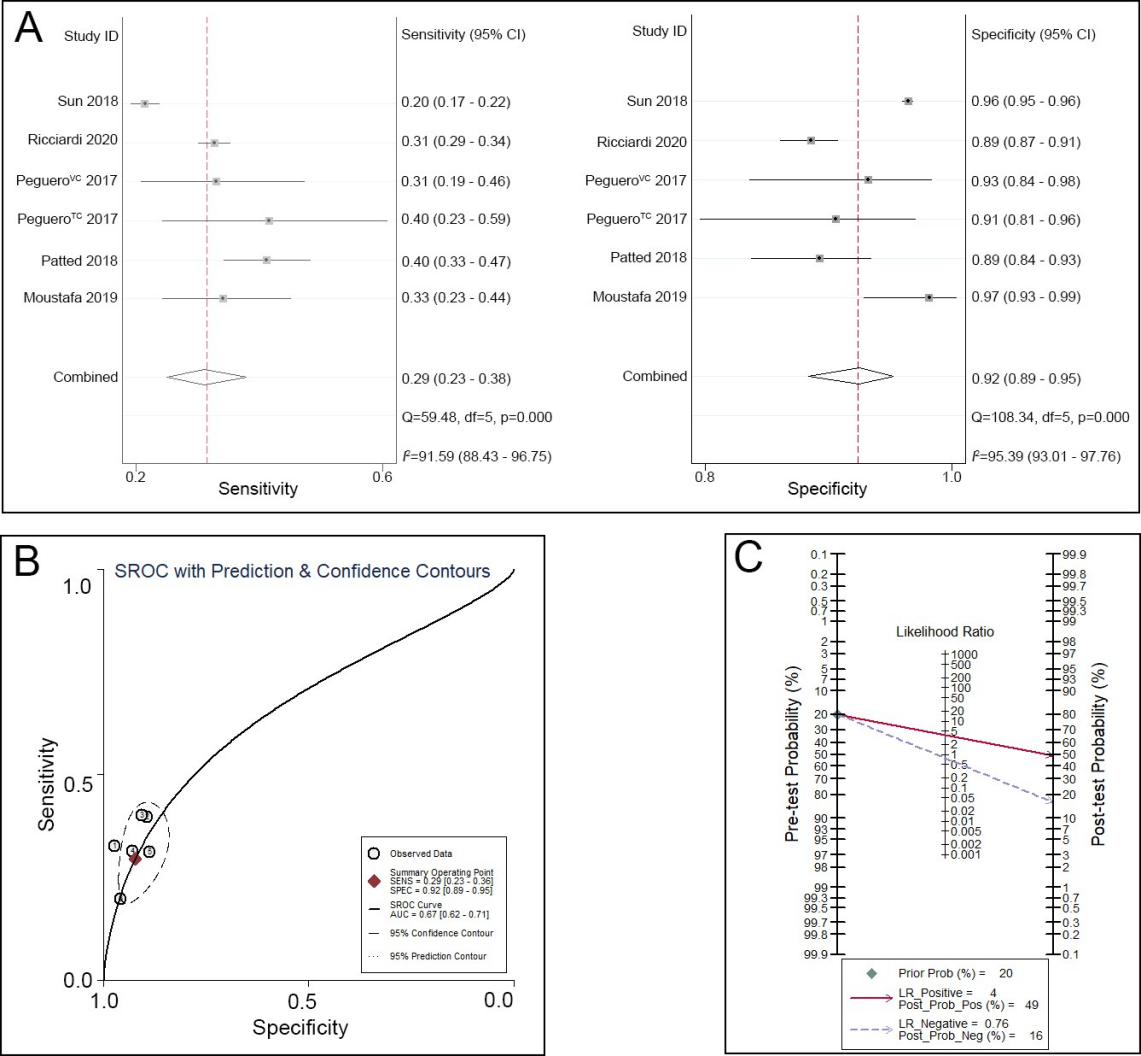
Cornell voltage index for the diagnosis of LVH. A. Sensitivity and specificity of the Cornell voltage index for the diagnosis of LVH. B. SROC curve of the Cornell voltage index for the diagnosis of LVH. C. Fagan nomogram of the Cornell voltage index for the diagnosis of LVH.

Regarding the Cornell voltage index, the sensitivity ranged from 0.20 to 0.40, and the specificity ranged from 0.89 to 0.97. A pooled analysis of the Cornell voltage index in the diagnosis of LVH showed that the pooled sensitivity was 0.29 (95% CI 0.23–0.36), the specificity was 0.92 (95% CI 0.89–0.95) (Fig 4A), and the AUC was 0.67 (95% CI 0.62–0.71) (Fig 4B). Given a pretest probability of 20%, the pooled PLR and NLR of the Cornell voltage index for the diagnosis of LVH were 4 and 0.76, respectively; the posttest probability for a positive test result was 49%, and the posttest probability for a negative test result was 16% (Fig 4C).

**Fig 5:**
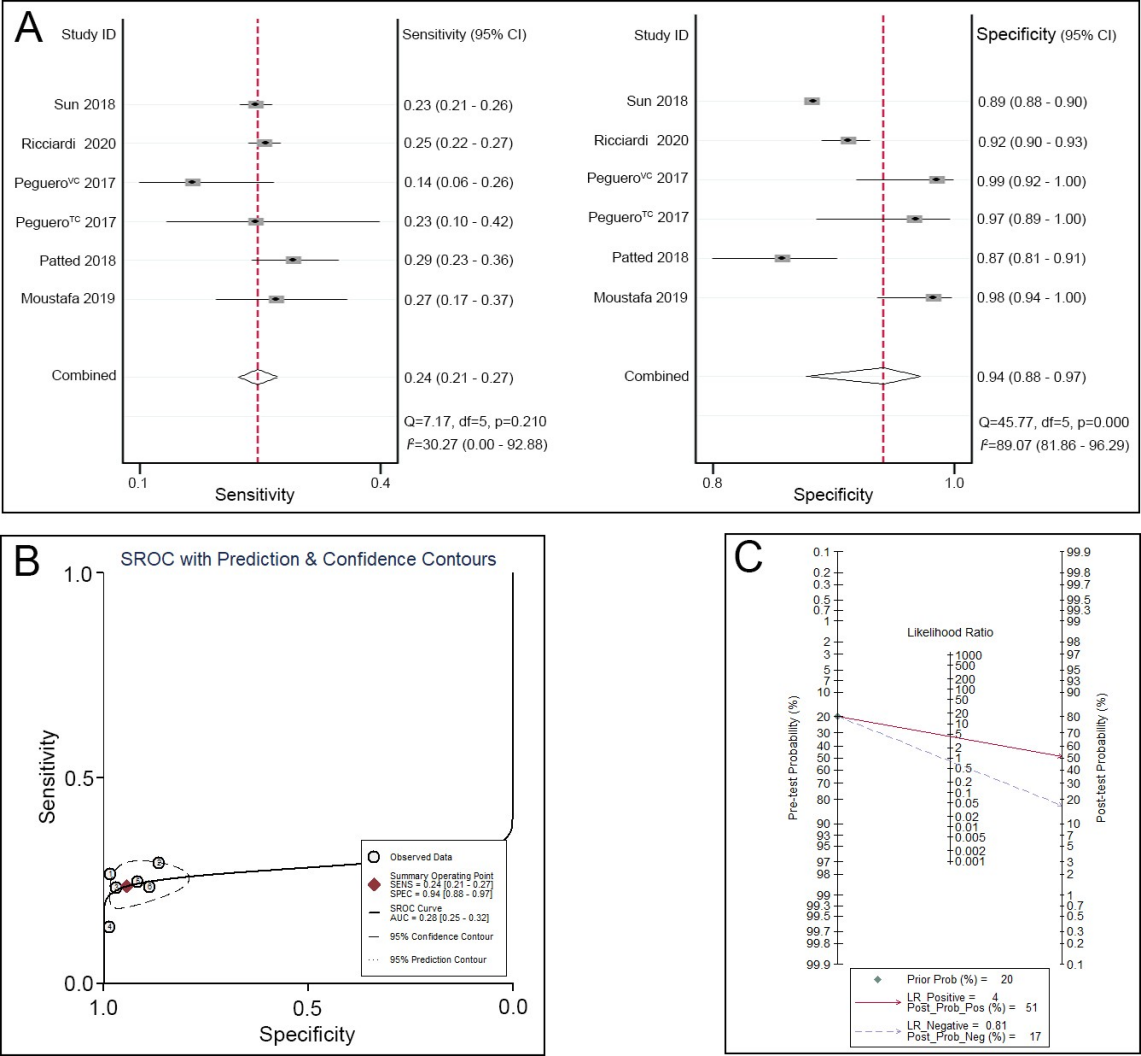
Sokolow-Lyon criteria for the diagnosis of LVH. A. Sensitivity and specificity of the Sokolow-Lyon criteria for the diagnosis of LVH. B. SROC curve of the Sokolow-Lyon criteria for the diagnosis of LVH. C. Fagan nomogram of the Sokolow-Lyon criteria for the diagnosis of LVH.

Regarding the Sokolow-Lyon criteria, the sensitivity ranged from 0.14 to 0.29, and the specificity ranged from 0.87 to 0.98. A pooled analysis of the Sokolow-Lyon criteria in the diagnosis of LVH showed that the pooled sensitivity was 0.24 (95% CI 0.21–0.27), the specificity was 0.94 (95% CI 0.88–0.97) (Fig 5A), and the AUC was 0.28 (95% CI 0.25–0.32) (Fig 5B). Given a pretest probability of 20%, the pooled PLR and NLR of the Sokolow-Lyon criteria for the diagnosis of LVH were 4 and 0.81, respectively; the posttest probability for a positive test result was 51%, and the posttest probability for a negative test result was 17% (Fig 5C).

However, substantial heterogeneity existed among the 6 included trials. Due to the same cutoffs being adopted in all included trials, the proportion of heterogeneity caused by the threshold effect was small. After carefully reading the full text of the included trials, we found that 2 trials conducted by Ricciardi et al. [18] and Sun et al. [16] included partly healthy subjects. After removing these 2 trials, the heterogeneity of the remaining 4 trials was small and acceptable [12–14].

**Fig 6:**
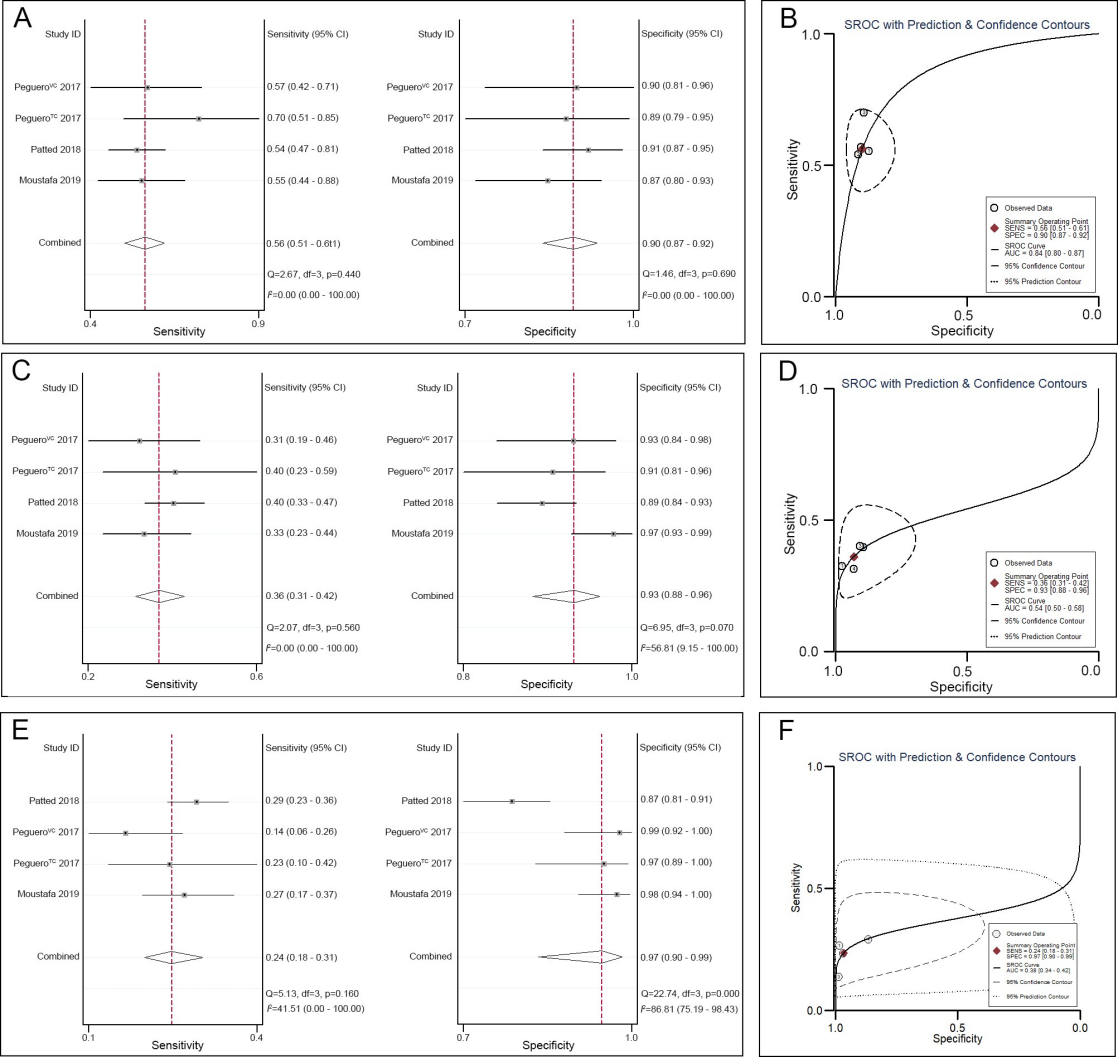
ECG criteria for the diagnosis of LVH. A. Sensitivity and specificity of the Peguero-Lo Presti criteria for the diagnosis of LVH. B. SROC curve of the Peguero-Lo Presti criteria for the diagnosis of LVH. C. Sensitivity and specificity of the Cornell voltage index for the diagnosis of LVH. D. SROC curve of the Cornell voltage index for the diagnosis of LVH. E. Sensitivity and specificity of the Sokolow-Lyon criteria for the diagnosis of LVH. F. SROC curve of the Sokolow-Lyon criteria for the diagnosis of LVH.

A pooled analysis of the Peguero-Lo Presti criteria in the diagnosis of LVH in the remaining 4 trials showed that the pooled sensitivity was 0.56 (95% CI 0.51–0.61), the specificity was 0.90 (95% CI 0.87–0.92) (Fig 6A), and the AUC was 0.84 (95% CI 0.80–0.87) (Fig 6B). A pooled analysis of the Cornell voltage index in the diagnosis of LVH in the remaining 4 trials showed that the pooled sensitivity was 0.36 (95% CI 0.31–0.42), the specificity was 0.93 (95% CI 0.88–0.96) (Fig 6C), and the AUC was 0.54 (95% CI 0.50–0.58) (Fig 6D). A pooled analysis of the Cornell voltage index in the diagnosis of LVH in the remaining 4 trials showed that the pooled sensitivity was 0.24 (95% CI 0.18–0.31), the specificity was 0.97 (95% CI 0.90–0.99) (Fig 6E), and the AUC was 0.38 (95% CI 0.34–0.42)) (Fig 6F).

## Discussion

In the last 3 years, the Peguero-Lo Presti criteria-based ECG diagnostic method for LVH has attracted increasing attention. Pooled data analysis showed that the Peguero-Lo Presti criteria-based ECG diagnostic method for diagnosing LVH has a high sensitivity, specificity and diagnostic accuracy.

LVH, which is caused by an increase in left ventricular mass, can be estimated by electrical voltage changes, which can be detected by 12-lead ECG [24–26]. The Cornell voltage index, which combines the projection of the cardiac cross sectional and frontal planes, can fully reflect the spatial vector of LVH and is the most commonly used ECG criterion [27–29]. Consistent with the results of previous studies, the results of this study also found that the Cornell voltage index had generally high specificity (92%) but poor sensitivity (29%). Similar results were also obtained for the Sokolow-Lyon criteria (specificity 94%, sensitivity 24%), another commonly used clinical ECG criteria of LVH that reflects the condition of LVH from the horizontal section of the heart [30]. In addition to the amount of myocardium and relative ventricular thickness, there are still many factors (e.g., fibrosis of the myocardium, chest wall thickness, distance of left ventricular cavity–electrode, individual conduction differences, electrical properties of the body, activity of the lung) that affect electrical voltage changes [17, 31–33]. All of these factors may lead to the fluctuation in the ECG voltage and attenuate the reproducibility of the ECG examination.

Based on the above theory, to improve the sensitivity of ECG to diagnose LVH, Peguero et al. proposed the novel Peguero-Lo Presti criteria (S_D_+SV_4_); the Peguero-Lo Presti criteria have been shown to have a better diagnostic performance, mainly in the sensitivity, than that in the Cornell voltage index [12]. This advantage has been demonstrated by numerous individual studies [13–15, 34, 35]. While being recognized as having good diagnostic performance by peers, there are many who still doubt the performance of the Peguero-Lo Presti criteria.

First, this criterion was developed and assessed from 2 cohorts (test and validation cohorts) in the American population with a relatively small sample size (94 and 122 patients, respectively). Therefore, the representation and reliability of the results are poor. The evidence is that professor Sun found the Peguero-Lo Presti criteria may not be a good screening tool for LVH in Asian populations [16]. They found that the diagnostic performance of this criterion was influenced by relative wall thickness [36]. Another disagreement is that the specificity of the Peguero-Lo Presti criteria was found to be exceptionally poor in younger persons because it is age- and sex-dependent [23].

Given these arguments, we conducted this study to test and validate the overall performance of this criterion. The pooled data analysis showed that compared with the Cornell voltage index, the Peguero-Lo Presti criteria-based ECG diagnostic method for LVH has a high sensitivity (36% vs 56%) and similar specificity (93% vs 90%). Moreover, the results of our study demonstrated that the Peguero-Lo Presti criteria have a high diagnostic accuracy (AUC = 0.84, 95% CI: 0.80–0.87). These results were consistent with the recent meta-analysis that proved that the Peguero-Lo Presti criteria had better diagnostic performance than the Cornell voltage index and the Sokolow-Lyon criteria and thus might be more useful in routine clinical practice as a screening tool for LVH [19]. Thus, measuring the amplitude of the deepest S wave (S_D_) in ECG leads could improve the diagnostic sensitivity and accuracy of LVH by ECG.

A Fagan nomogram was adopted to calculate the likelihood ratios and posttest probabilities, which provide important information about the likelihood that a patient with a positive or negative test actually has LVH or not [37]. In this study, both the likelihood ratio and posttest probability of the Peguero-Lo Presti criteria and Cornell voltage index were moderate. Given a pretest probability of 20%, the posttest probability of the Peguero-Lo Presti criteria and Cornell voltage index were similar.

## Study limitations

In this meta-analysis, several limitations should be noted. First, we detected substantial heterogeneity caused by primary disease in the included subjects among the included trials. After removing the 2 trials that included partly healthy subjects, the heterogeneity of the remaining 4 trials, which included subjects with different kinds of cardiac disease, was small and acceptable. Thus, the results of this study would be limited to the population with different kinds of cardiac disease. In other words, the Peguero-Lo Presti criteria might not be appropriate as a screening tool for LVH in community populations.

Second, the reference standard of LVH was defined by LVMI, which was calculated and estimated by using echocardiography. Although echocardiography has good reproducibility for the diagnosis of LVH, echocardiography ignores LVH that occurs in the initial stages [38]. Because the LVMI value of this study was >115 g/m^2^ in male subjects and >95 g/m^2^ in female subjects, we did not include a trial that adopted different LVMI values (>102 g/m^2^ in male subjects and >88 g/m^2^) [17]. We also did not include a trial that adopted cardiac magnetic resonance as the reference standard for LVH diagnosis [15]. All of these factors may lead to different degrees of selection bias.

Third, we included only 6 trials, so a publication bias was not detected in this meta-analysis. Moreover, half of the included trials had a small sample size (less than 200 patients) [12, 13]. Fourth, we did not collect any unpublished data. Finally, a total of 6 (2.78%) atrial fibrillation patients were included in 2 trials [12]. This would undoubtedly decrease the accuracy of an ECG in diagnosing LVH.

## Conclusions

The Peguero-Lo Presti criteria-based ECG diagnostic method for LVH has a high sensitivity, specificity and diagnostic accuracy and should be applied in clinical practice settings. However, given these limitations, a large sample size and strict design trials are necessary in further studies.

## Supporting information

S1 Table PRISMA 2009 checklist. (DOCX)

S2 Table Electronic search terms. (DOCX)

S3 Table Characteristics of the included trials. (DOCX)

S4 Table History of heart disease and medications in the included trials. (DOCX)
