# Supplementary material for: Peguero-Lo Presti criteria for the diagnosis of left ventricular hypertrophy: A systematic review and meta-analysis
Source: PLoS One. 2021 Jan 29;16(1):e0246305. doi: 10.1371/journal.pone.0246305 (PMC7846009; doi:10.1371/journal.pone.0246305)
Supplement: S2 Table — (DOCX) [file pone.0246305.s002.docx]

**S2 Table.** Electronic search terms

| MEDLINE | #1 "Hypertrophy, Left Ventricular"[Mesh]  #2 "Left Ventricular Hypertrophy"[All Fields]  #3 "Hypertrophies, Left Ventricular"[All Fields]  #4 "Ventricular Hypertrophies, Left"[All Fields]  #5 "Ventricular Hypertrophy, Left"[All Fields]  #6 "LVH"[All Fields]  #7 #1 OR #2 OR #3 OR #4 OR #5 OR #6  #8 "Peguero Lo-Presti"[All Fields]  #9 #7 AND #8 |
| --- | --- |
| Web of Science | #1 'Hypertrophy, Left Ventricular':ab,ti  #2 'Left Ventricular Hypertrophy':ab,ti  #3 'Hypertrophies, Left Ventricular':ab,ti  #4 'Ventricular Hypertrophies, Left':ab,ti  #5 'Ventricular Hypertrophy, Left':ab,ti  #6 'LVH':ab,ti  #7 #1 OR #2 OR #3 OR #4 OR #5 OR #6  #8 'Peguero Lo-Presti':ab,ti  #9 #7 AND #8 |
| Embase | #1 'left ventricle hypertrophy'/exp  #2 'Hypertrophy, Left Ventricular'  #3 'Hypertrophies, Left Ventricular'  #4 'Ventricular Hypertrophies, Left'  #5 'Ventricular Hypertrophy, Left'  #6 'LVH'  #7 #1 OR #2 OR #3 OR #4 OR #5 OR #6  #8 'Peguero Lo-Presti'  #9 #7 AND #8 |
| Cochrane library | #1 MeSH descriptor: [Hypertrophy, Left Ventricular] explode all trees  #2 left ventricle hypertrophy :ti,ab,kw (Word variations have been searched)  #3 Hypertrophies, Left Ventricular :ti,ab,kw (Word variations have been searched)  #4 Ventricular Hypertrophies, Left :ti,ab,kw (Word variations have been searched)  #5 Ventricular Hypertrophy, Left :ti,ab,kw (Word variations have been searched)  #6 LVH :ti,ab,kw (Word variations have been searched)  #7 #1 OR #2 OR #3 OR #4 OR #5 OR #6  #8 Peguero :ti,ab,kw (Word variations have been searched)  #9 #7 AND #8 |
