# Supplementary material for: Peguero-Lo Presti criteria for the diagnosis of left ventricular hypertrophy: A systematic review and meta-analysis
Source: PLoS One. 2021 Jan 29;16(1):e0246305. doi: 10.1371/journal.pone.0246305 (PMC7846009; doi:10.1371/journal.pone.0246305)
Supplement: S3 Table — (DOCX) [file pone.0246305.s003.docx]

**S3 Table. Characteristics of included trials.**

| **Name** | **Year** | **Design** | **Inclusion Criteria** | **Exclusion Criteria** | **Follow-up Period** |
| --- | --- | --- | --- | --- | --- |
| Moustafa **^13^** | 2019 | Observational cross sectional study | 1. Patients with history of CAD.  2. Presence of angiographically proven CAD. | 1. Reduced LV EF on echocardiography (EF< 50%);  2. Bundle branch block;  3. Valvular heart disease;  4. Congenital heart disease;  5. Constrictive cardiomyopathy;  6. Hypertrophic cardiomyopathy;  7. Atrial fibrillation or flutter;  8. Wolff Parkinson-White syndrome. | From July 2016  to June 2017 |
| Patted **^14^** | 2018 | Cross-sectional study | 1. Age above 30 years.  2. Presenting with hypertension. | 1. Myocardial infarction;  2.Valvular heart disease (Grade II or higher);  3.Valvular stenosis;  4. LV dysfunction;  5. Pericardial disease;  6. COPD;  7.Bundle branch blocks;  8. Atrial fibrillation or flutter. | From May 2017  to December 2017 |
| Peguero^TC^ **^12^** | 2017 | Retrospective design | Cardiovascular disease. | 1. Complete left or right bundle branch block.  2. Ventricular paced rhythm.  3. Poor echocardiographic windows. | From August  to September 2013. |
| Peguero^VC^ **^12^** | 2017 | Retrospective design | Cardiovascular disease. | 1. Complete left or right bundle branch block.  2. Ventricular paced rhythm.  3. Poor echocardiographic windows. | From January 2014  to February 2014. |
| Ricciardi **^18^** | 2020 | Retrospective design | 1. All patients who underwent a 12-lead ECG and a transthoracic echocardiogram.  2. Regardless of their initial admitting diagnosis. | 1. Bundle-branch block.  2. Left anterior fascicle block.  3. Paced rhythm.  4. Pathological Q waves. | From January 2012  to December 2017 |
| Sun **^16^** | 2018 | Retrospective design | 1. Permanent residents.  2. Age ≥ 35 years. | 1. Poor echocardiographic or ECG quality;  2. Complete left or right bundle branch block;  3. Ventricular paced rhythm  4. Pregnant Cancer, mental disorders.  5. Missing data | From January 2013  to August 2013. |

CAD: coronary artery disease; LV: left ventricular; EF: ejection fraction; ECG: electrocardiogram; COPD: chronic obstructive pulmonary disease; TC: test cohort; VC: validation cohort;
