# Supplementary material for: Peguero-Lo Presti criteria for the diagnosis of left ventricular hypertrophy: A systematic review and meta-analysis
Source: PLoS One. 2021 Jan 29;16(1):e0246305. doi: 10.1371/journal.pone.0246305 (PMC7846009; doi:10.1371/journal.pone.0246305)
Supplement: S4 Table — (DOCX) [file pone.0246305.s004.docx]

**S4 Table. History of heart disease and medication of included trials**

| **Name** | **Publication**  **year** | **Size**  **(n)** | **History**  **of MI** | **Previous**  **CABG** | **Previous**  **PCI** | **Use of**  **beta-blockers** | **Use of**  **ACEI/ARBs** | **Use of calcium**  **channel blockers** |
| --- | --- | --- | --- | --- | --- | --- | --- | --- |
| Moustafa **^13^** | 2019 | 200 | 53(26.50) | 44(22.00) | 152(76.00) |  |  |  |
| Peguero^TC^ **^12^** | 2017 | 94 | 10(10.64) | 3(3.19) | 8(8.51) | 34(36.17) | 36(38.30) | 24(25.53) |
| Peguero^VC^ **^12^** | 2017 | 122 | 11(9.02) | 7(5.74) | 10(8.20) | 49(40.16) | 55(45.08) | 26(21.31) |

TC: test cohort; VC: validation cohort; MI: myocardial infarction; CABG: coronary artery bypass graft; PCI: percutaneous coronary intervention; ACEI: angiotensin converting enzyme inhibitors; ARBs: angiotensin receptor blockers.
